# Betulinic acid triggers apoptosis and inhibits migration and invasion of gastric cancer cells by impairing EMT progress

**DOI:** 10.1002/cbf.3537

**Published:** 2020-04-13

**Authors:** Yun Chen, Xiongjian Wu, Chi Liu, Yun Zhou

**Affiliations:** ^1^ Digestive System Department First Affiliated Hospital of Gannan Medical University Ganzhou People's Republic of China; ^2^ School of Medical & Life Sciences Chengdu University of Traditional Chinese Medicine Chengdu People's Republic of China

**Keywords:** apoptosis, betulinic acid, epithelial‐mesenchymal transition, gastric cancer, migration

## Abstract

Gastric cancer (GC) is one of the most prevalent types of malignancies. Betulinic acid (BA) is a natural pentacyclic triterpene with a lupine structure. However, to the best of our knowledge, there is no research study on the anti‐tumour and anti‐metastasis effect of BA on GC. In this study, we assessed the anti‐cancer effect of BA on human GC cells in vitro and in vivo. We first investigated the cytotoxicity and anti‐proliferation effect of BA on GC cells of SNU‐16 and NCI‐N87. The results indicated that BA had significant cytotoxic and inhibitory effects on GC cells in a dose‐ and time‐dependent manner. To further study the cytotoxic action of BA on GC cells, we assessed the apoptotic induction effect of BA on SNU‐16 cells and found that BA distinctly induced apoptosis in SNU‐16 cells. In addition, BA inhibited the migratory and invasive abilities of SNU‐16 cells. Western‐blot analysis revealed that BA suppressed the migration and invasion of GC cells by impairing epithelial‐mesenchymal transition progression. Furthermore, in vivo experiments showed that BA could delay tumour growth and inhibit pulmonary metastasis, which is consistent with the results of in vitro studies. Overall, we evaluated the anti‐cancer effect of BA on human GC cells in vivo and in vitro, and the present study provides new evidence on the use of BA as a potential anti‐cancer drug for GC treatment.

**Significance of the study:**

BA significantly suppressed proliferation and triggered apoptosis in GC cells. Additionally, BA remarkably inhibited migration and invasion of GC cells by impairing the epithelial‐mesenchymal transition signalling pathway. It is worth noting that BA drastically retarded tumour growth in the xenograft mouse model of GC. Our results indicated that BA can be considered a candidate drug for GC therapy.

## 1 INTRODUCTION

Gastric cancer (GC) is one of the most prevalent types of malignancies. More than 1.3 million cases of GC and 819 000 GC‐associated cases of mortality were reported worldwide in 2015, which makes GC the third leading cause of cancer‐associated mortality.^1^ GC plays an important role in tumour‐related mortality worldwide, especially in Asian countries.^2^ Stomach cancer has the second highest tumour‐related mortality in China.^3^ Distant migration and invasion of GC cells is the main cause of mortality from GC.^4^, ^5^, ^6^ Most patients have metastases at the time of diagnosis, and their 5‐year survival rates are extremely low.^7^ In 2018, 679 000 new cases and 498 000 cases of GC‐related mortality were reported by the Chinese National Cancer Center.^8^ With the use of multimodal treatments for GC, the overall 5‐year survival rate has been found to stabilize at 40% worldwide.^9^, ^10^, ^11^ Therefore, it is crucial to understand the molecular mechanism of GC progression and metastasis in early diagnosis and treatment. Currently, modern treatment of GC, including surgery combined with radiotherapy, chemotherapy and targeted therapy still have some drawbacks such as low therapeutic effect, high toxicity, recurrence and even metastasis.^12^, ^13^ Therefore, it is important to find safe and effective drug candidates for GC treatment.

Natural products have been used to treat human diseases since ancient times and are they are vital to drug discovery and development.^14^, ^15^ Various anti‐infection and anti‐cancer drugs have been derived from natural products.^16^, ^17^, ^18^, ^19^ Additionally, the rapid development of new drugs that are more effective and have fewer adverse effects is a common goal shared by scientists and clinicians.^20^, ^21^ Traditional Chinese medicine is a huge treasury of remedies with low toxicity and high sensitivity and the ability to stabilize tumour growth and improve GC prevention and treatment.

**FIGURE 1:**
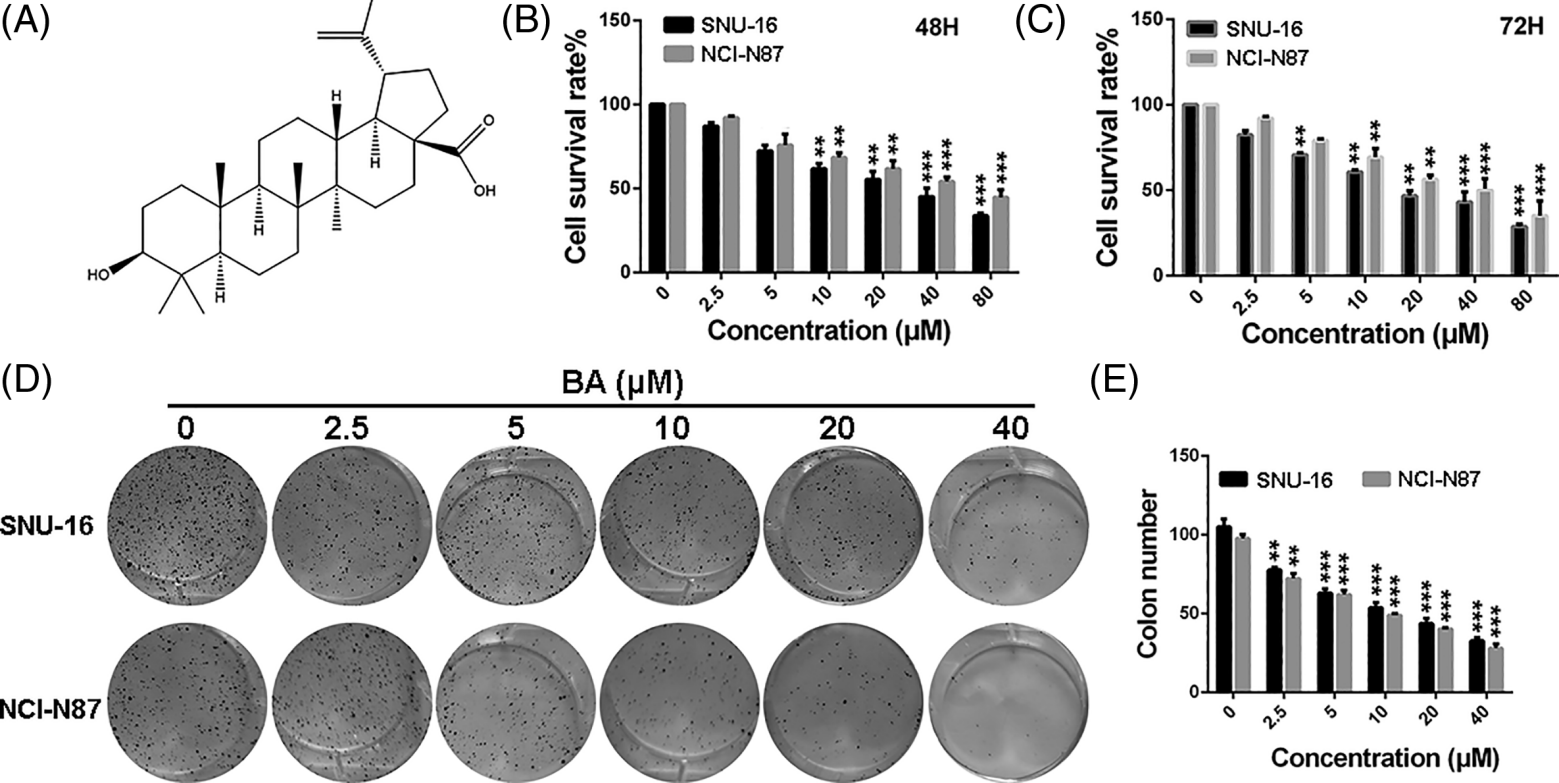
BA inhibits viability and proliferation of gastric cancer cells. A, The chemical structure of BA. B and C, The growth curves of SNU‐16 and NCI‐N87 cells treated with different concentrations of BA for 48 and 72 hours. D and E, The colony formation of SNU‐16 and NCI‐N87 cells treated with different concentrations of BA. Significant differences were indicated as *P ≤ .05; **P ≤ .01; ***P ≤ .001. B, betulinic acid

Betulinic acid (BA) (Figure 1A) (3β, hydroxyl‐lup‐20[29]‐en‐28‐oic acid) is a natural pentacyclic triterpene with a lupine structure, which is derived from plant sources such as acuminatissima leaves, white birch bark and wild jujube seeds.^22^, ^23^ Various studies have reported that BA has a variety of valuable medicinal effects, including anti‐bacterial, anti‐cancer, anti‐malarial, anti‐viral and anti‐inflammatory.^24^, ^25^, ^26^ Furthermore, BA has shown tumorigenesis inhibition in many kinds of cancers, including lung, colon, breast, prostate and pancreatic cancer.^27^, ^28^, ^29^, ^30^ In the present study, our results showed that BA distinctly triggered apoptosis and suppressed the proliferation, migration and invasion of GC cells in vitro. The in vivo anti‐tumour efficacy was consistent with that reported in vitro studies.

## 2 MATERIALS AND METHODS

### 2.1 Reagents and antibodies

BA was purchased from Zhejiang Haizheng Pharmaceutical Co, Ltd (Hangzhou, China). BA was initially prepared as an 80‐mM stock solution in dimethyl sulfoxide (DMSO; Sigma‐Aldrich, St Louis, MO) and stored at −20°C for further use in in vitro experiments. Determined concentrations (0, 2.5, 5, 10, 20, 40 and 80 μM) of BA were prepared by the diluting stock solution with cell culture medium. Solutions containing 0.1% DMSO were regarded the control group in vitro. For animal studies, BA was dissolved in 40% (vol/vol) polyethylene glycol with 5% (vol/vol) DMSO. The primary antibodies of E‐cadherin (#14472), N‐cadherin (#13116), Ki‐67 (#9449), MMP‐2 (#40994) and β‐actin (#4970) were purchased from Cell Signalling Technology (Beverly, Massachusetts).

### 2.2 Cell lines and cell culture

SNU‐16 and NCI‐N87 cells were purchased from the American Type Culture Collection (ATCC). Cells were cultured in RPMI1640 medium (Gibco, Grand Island, New York) containing 10% fetal bovine serum (FBS) (Gibco, Grand Island, New York). All cells were kept in an atmosphere of 5% CO_2_ at 37°C.

### 2.3 Cell viability assays

Cell viability was assessed by the 3‐(4,5‐dimethylthiazol‐2‐yl)‐2,5‐diphenyltetrazolium bromide (MTT) method. Briefly, SNU‐16 and NCI‐N87 cells (2 × 10^3^) that were seeded in the wells of a 96‐well plate and cultured for another 12 hours, were exposed to determined concentrations of BA (0, 2.5, 5, 10, 20, 40 and 80 μM) for 48 and 72 hours, respectively. Then, the supernatant of the culture medium was discarded and 20 μL of MTT (5 mg/mL) was added. After incubation for another 4 hours at 37°C, MTT solution was substituted by 150 μL of DMSO (Sigma‐Aldrich). The absorbance of each well were recorded at 570 nm by a microplate reader (Bio‐Tek Instruments, Bad Friedrichshall, Germany).

### 2.4 Colony‐formation assays

Approximately 500 GC cells were seeded into a six‐well plate and cultured in media with various concentrations of BA (0, 2.5, 5, 10, 20, 40 and 80 μM). The cells were incubated at 37°C for 8 days. When larger clones were found in the control group, the incubation was stopped. The culture medium was discarded, and cells were washed with phosphate‐buffered saline (PBS), fixed with 800 μL methanol per well for 10 to 20 minutes, and stained with 0.1% crystal violet at 37°C for 10 minutes. Finally, the stained cell clones in dishes were imaged and counted. Each treatment was repeated in three times.

### 2.5 Morphological analysis of nuclei by Hoechst staining

Apoptotic cells have obvious morphologic characteristics: cell body shrinkage, chromatin condensation, margination and emerging apoptotic bodies. To determine whether BA triggered apoptosis in GC cells, we stained the SNU‐16 cells with Hoechst 33258 dye according to the manufacturer's instructions. Briefly, SNU‐16 cells (1 to 2 × 10^5^ cells/well) were seeded into six‐well plate and cultured for 12 hours. After treatment with different concentrations (0, 10, 20 and 40 μM) of BA for 48 hours, the cells were washed with ice‐cold PBS three times and fixed with methanol for 20 minutes. Then, the nuclear morphology of the apoptotic cells was photographed by a fluorescence microscope (Leica, DM4000B).

### 2.6 Cell apoptosis analysis

Cell apoptosis was assessed by Annexin V/PI dual staining via flow cytometry (FCM). In brief, the cells were seeded into a six‐well plate and treated with determined concentrations of BA (0, 10, 20 and 40 μM) for 48 hours. Then, the cells were harvested, washed with PBS and labelled with Annexin V/PI apoptotic analysis kits (BD) and analysed by FCM. The sum of late and early apoptosis (Annexin V^+^/PI^+^ and Annexin V^+^/PI^−^) was regarded as the apoptosis rate in this study.

### 2.7 Transwell migration and invasion assays

For the cell migration analysis, 2 × 10^4^ SNU‐16 cells suspended in FBS‐free culture medium containing different concentrations of BA (0, 10, 20 and 40 μM) were seeded into the upper chamber of a transwell, and 600 μL of medium containing 10% FBS were added to the lower chamber. Then, the cells were cultured for 48 hours in a humidified incubator with 5% CO_2_ at 37°C. The migrated cells were fixed with methanol, stained with 0.5% crystal violet and counted under a microscope. For the cell invasion experiment, the upper chamber was pre‐treated with Matrigel (BD). Subsequent procedures were consistent with the cell migration experiment. Data are represented as rate of migration and invasion in comparison with the untreated group.

### 2.8 Western blot analysis

Total protein was separated from the treated cells using 500 μL of radio immune precipitation assay buffer with 1 mM of phenylmethanesulfonylfluoride. Samples with the cells were immediately sonicated for 2 minutes to break the cell membrane, and were then centrifuged. The supernatant containing the proteins was collected and stored at −20°C for further use. The proteins were separated by 10% polyacrylamide gels and then transferred onto a polyvinylidene fluoride membrane. Then, the proteins on the membranes were washed with TBS containing 0.1% Tween‐20 (TBST) and blocked by 5% nonfat milk (wt/vol) for 1 hour at room temperature. After washing with PBST three times, the membranes were incubated with antibodies (E‐cadherin, N‐cadherin and β‐actin) overnight at 4°C. Next, the membranes were washed again with PBST and incubated with horseradish peroxidase‐labelled immunoglobulin G (dilution, 1:5000) at room temperature for 1 hour. Finally, the proteins bands were visualized using enhanced chemiluminescence by western blot analysis.

### 2.9 Xenograft tumour model in nude mice

The animal experiment in this study was approved by the Institutional Animal Care and Use Committee of First Affiliated Hospital of Gannan Medical University. Male BALB/c nude mice (8 weeks old) were bought from Beijing HFK Bioscience Co, Ltd (Beijing, China). The mice were randomly divided into two groups (three mice per group) and kept in barrier facilities with rodent chow and water under a specific pathogen‐free condition. SNU‐16 cells (1 × 10 ^7^ cells/mouse) which were suspended in 100 μL of RPMI‐1640 medium were subcutaneously injected into the flanks of the mice. Four days later, the mice were administered various concentrations of BA (control and 40 mg/kg) by gavage once daily for 21 days. The mice in the control group were administered corresponding amount of vehicle (40% [vol/vol] polyethylene glycol with 5% [vol/vol] DMSO). Tumour volume and body weight of the mice were recorded every 3 days after treatment. The tumour volume was calculated according to the following formula: tumour volume (mm^3^) = L × W^2^/2, where L is length of the tumour and W is width. All mice were euthanized at day 21 after treatment, and their tumours and lungs were isolated, weighed and imaged. To establish a pulmonary metastasis model, a total of 1 × 10^6^ SNU‐16 cells were injected into the tail vein of the BALB/c mice. Three days after inoculation, the mice were randomized into two groups (n = 3), control group and group of 40 mg/kg, for further administration of BA once daily for 14 days. At the end of the experiment, the mice were killed. Lung tissues were isolated and weighted, and metastatic tumour nodules in lung tissues were counted under a dissecting microscope. For haematoxylin and eosin (H&E) analysis, the tumours were collected, fixed in 4% paraformaldehyde, embedded in paraffin and sectioned (5 μm thickness). After dewaxing and rehydration, the sections were stained with H&E at room temperature for 10 seconds and imaged under a light microscope (Olympus, Tokyo, Japan).

### 2.10 Immunohistochemistry

The tumour samples were resected from the mice, fixed in 10% formaldehyde, embedded in paraffin, and sectioned (5 μm thickness). The paraffin tumour sections were analysed by Immunohistochemistry (Ki‐67 and MMP‐2). Then, the sections were dehydrated and fixed, and the slides were sealed with neutral gum. Pictures were obtained using the Leica microscope (Leica, DM4000B).

### 2.11 Statistical analysis

All experiments were conducted at least three times. Data are showed as mean ± SD (SD). Two‐tailed student's *t* test and one‐way analysis of variance (ANOVA) test were used for statistical analysis of the data. Significant differences of the *P*‐values are as follows: * *P* < .05; ** *P* < .01; *** *P* < .001.

## 3 RESULTS

### 3.1 Anti‐proliferation effect of BA in human GC cells

For examining the potential pharmacological effect of BA on human GC cells, we assessed the cytotoxicity of BA on GC cells by exposing GC cell lines of SNU‐16 and NCI‐N87 to different concentrations (0, 2.5, 5, 10, 20, 40 and 80 μM) of BA for 48 and 72 hours, respectively. As displayed in Figure 1B,C, BA gradually inhibited the proliferation of SNU‐16 and NCI‐N87 cells as the drug concentration and treatment time increased. These data indicate that BA suppressed the viability of GC cells in a concentration‐ and time‐dependent manner. Furthermore, colony‐formation assays were carried out to study the anti‐proliferation effect of BA on SNU‐16 and NCI‐N87 cells. As shown in Figure 1D,E, the colony‐formation ability of SNU‐16 and NCI‐N87 cells was significantly inhibited by BA treatment. Taken together, the results show that BA exerted sufficient anti‐proliferation effect on GC cells.

### 3.2 BA triggers apoptosis in human GC cells

**FIGURE 2:**
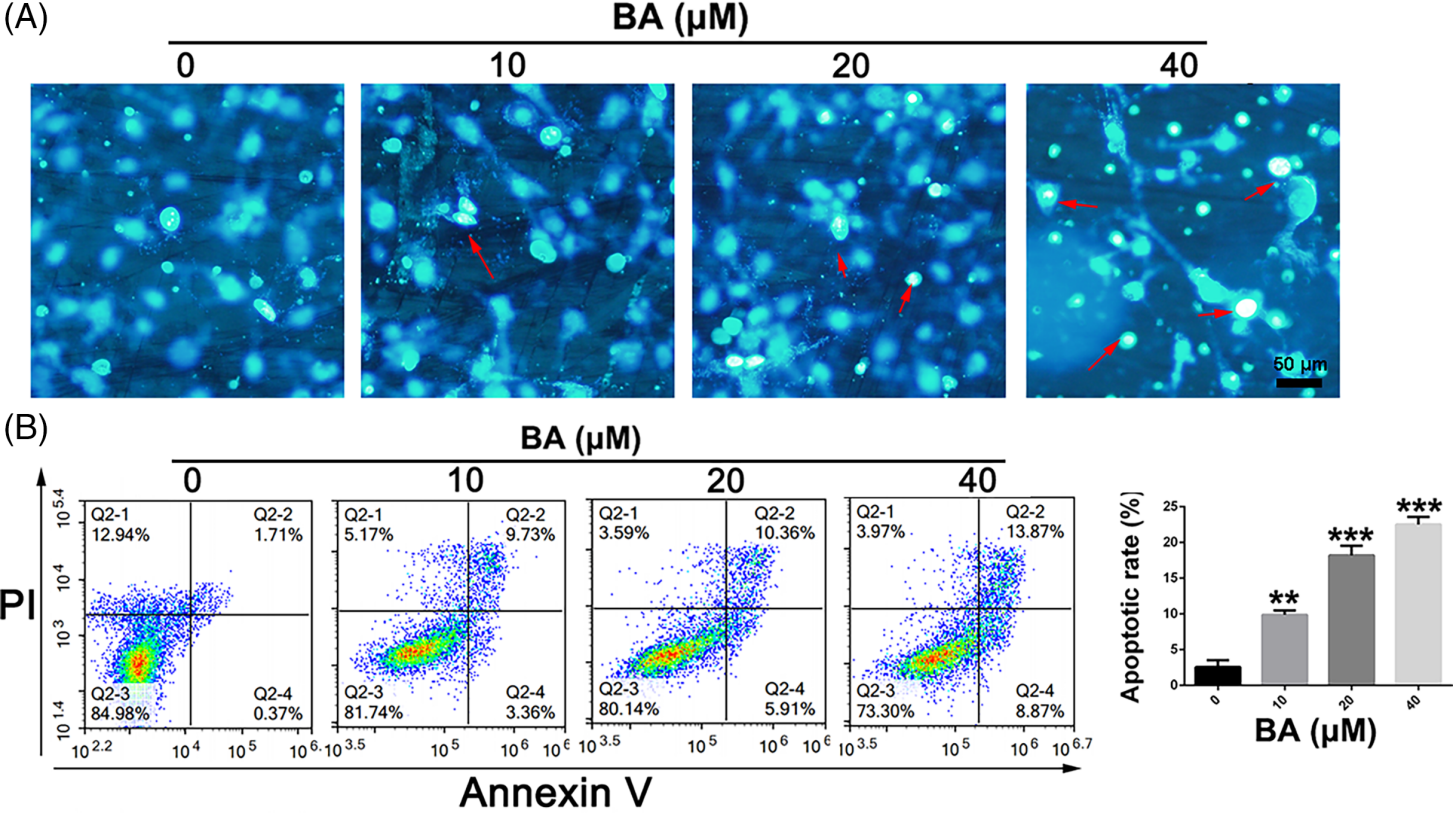
BA induces apoptosis in gastric cancer cells. A, Apoptosis of SNU‐16 cells treated with different concentrations of BA was analysed by Hoechst33258 staining. B, Apoptosis of SNU‐16 cells treated with different concentrations of BA was analysed by Annexin V/PI dual staining. Significant differences were indicated as *P ≤ .05; **P ≤ .01; ***P ≤ .001. BA, betulinic acid

For exploring whether BA triggers GC cell apoptosis, Hochest33258 staining and Annexin V/PI dual staining FCM were conducted. As shown in Figure 2A, after treatment with various concentrations of BA, karyopyknosis and apoptotic bodies were obviously observed in SNU‐16 cells. In addition, compared with control group, BA treatment significantly induced dose‐dependent apoptosis in SNU‐16 cells. Therefore, BA could remarkably trigger apoptosis in SNU‐16 cell a dose‐dependent manner.

### 3.3 BA inhibits migration and invasion of human GC cells via EMT signalling pathway

**FIGURE 3:**
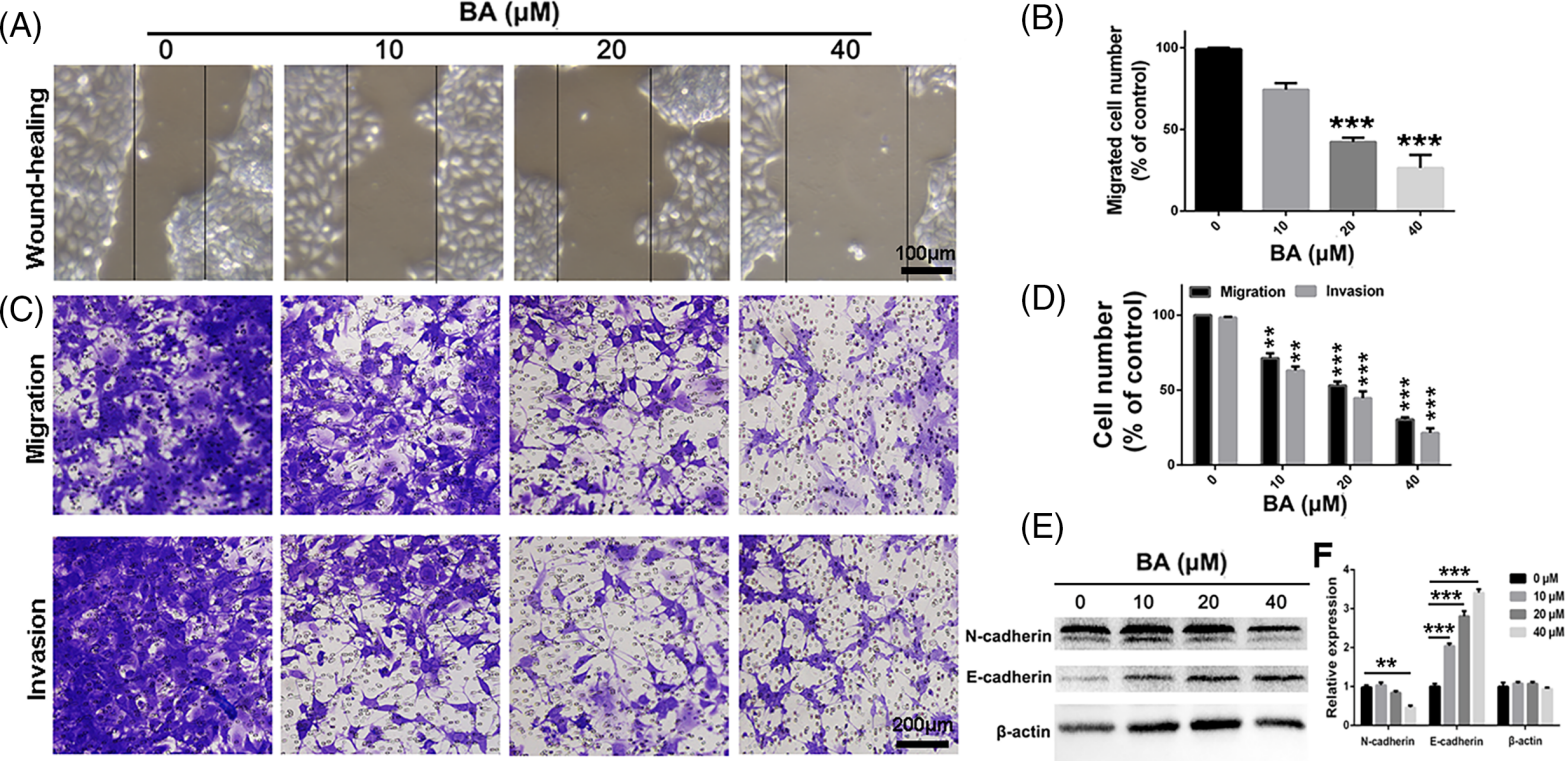
BA suppresses migration and invasion in gastric cancer cells. A and B, Wound healing assay was used to evaluate migration of SNU‐16 cells after treatment with different concentrations of BA. C and D, Transwell migration and invasion assay were exploited to assess migration and invasion of SNU‐16 cells after treatment with different concentrations of BA. E and F, The variation of epithelial‐mesenchymal transition associated metastatic proteins in SNU‐16 cells were analysed by western‐blot after treatment with different concentrations of BA. Significant differences were indicated as *P ≤ .05; **P ≤ .01; ***P ≤ .001. BA, betulinic acid

The migration, invasion and epithelial‐mesenchymal transition (EMT) of cancer cells are the important and key steps in the distant metastatic cascade of cancer cells. Transwell migration and invasion assays were conducted to study whether BA has an impact on the mobility of SNU‐16 cells after treatment with various concentrations of BA. As shown in Figure 3A,B, treatment with BA significantly impaired the mobility of SNU‐16 cell. Additionally, the migration and invasion activities of SNU‐16 cells were remarkably suppressed after treatment with BA (Figure 3C,D). To further study the intrinsic mechanisms of anti‐migration and anti‐invasion effects of BA on GC cells, the variation in the expression of metastatic proteins was analysed by western blot analysis. As displayed in Figure 3E,F, after treatment with BA, the relative expression level of N‐cadherin in SNU‐16 cells was drastically down‐regulated, and the expression of E‐cadherin in SNU‐16 cells was distinctly up‐regulated in comparison to that in the control group, implying a break in the EMT process. Therefore, the data suggest that BA represses migration and invasion activities of human GC cells via the EMT signalling pathway.

### 3.4 Anti‐tumour and anti‐metastasis effect of BA in a tumour xenograft model of SNU‐16 GC cells

**FIGURE 4:**
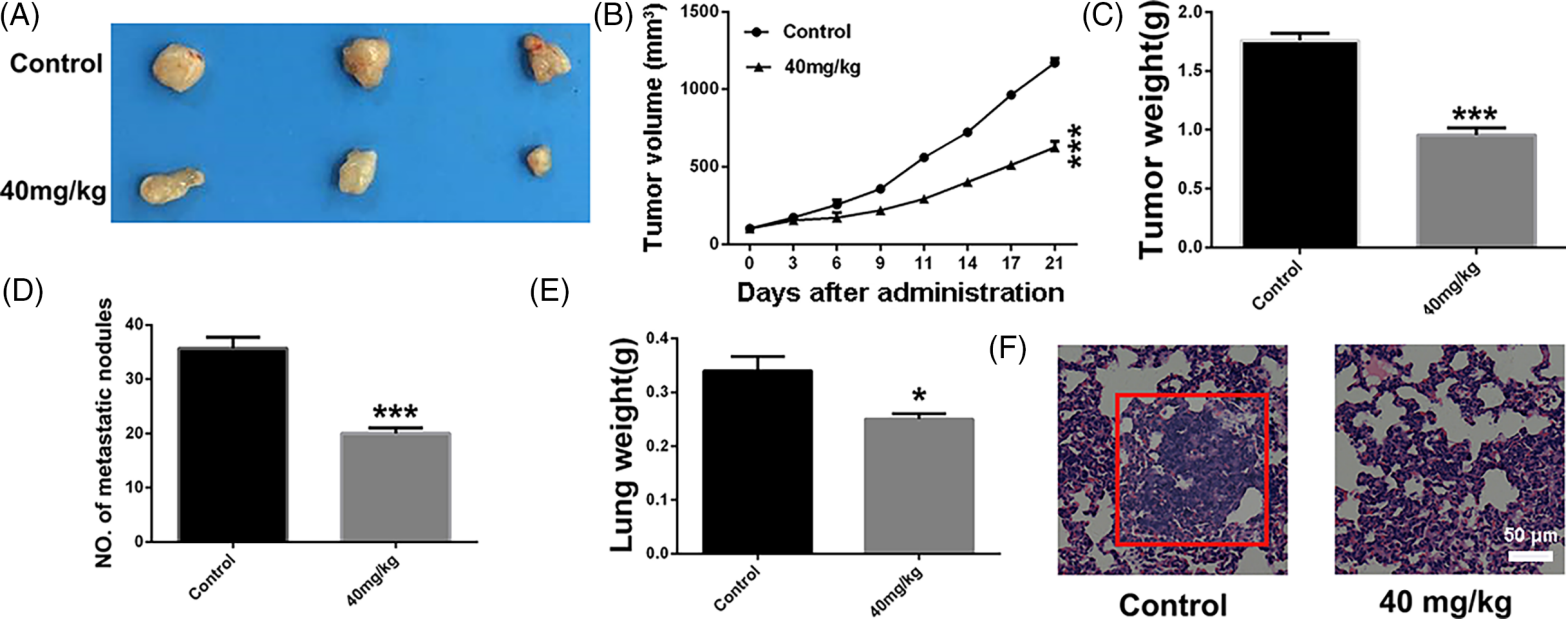
Betulinic acid inhibits gastric cancer cells growth and pulmonary metastasis in vivo. A, Representative images of tumour in different groups after different treatment at 21 days. B, Curve of tumour growth in the process of treatment. C, Tumour weight in different groups after different treatment at 21 days. D, Number of metastasis nodules in lungs of different treated groups. E, The weight of lungs from different treated groups. F, H&E analysis of lung sections of different treated groups. Significant differences were indicated as *P ≤ .05; **P ≤ .01; ***P ≤ .001

**FIGURE 5:**
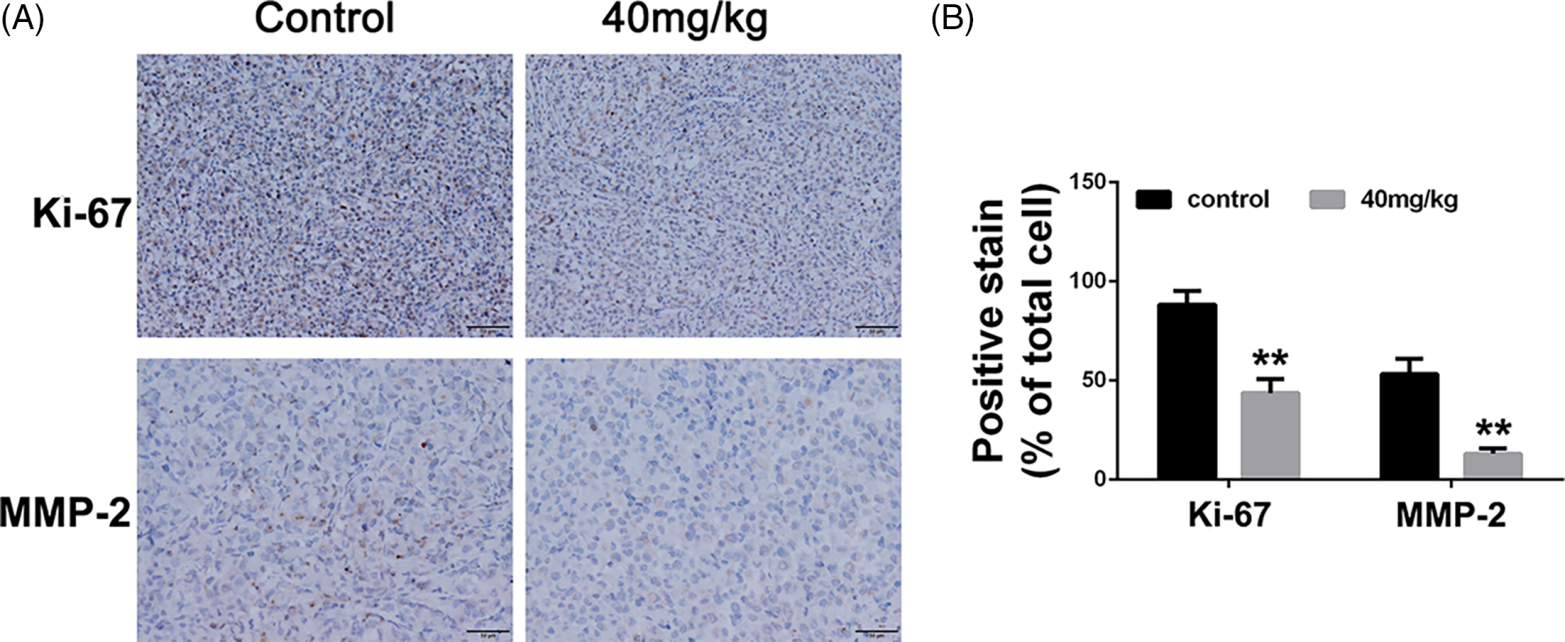
Betulinic acid inhibits proliferation and metastasis in gastric cancer cells tumour. A, Immunohistochemical analysis of Ki‐67 and MMP‐2 of tumour sections from different treated groups. B, Statistical analysis of immunohistochemical results of Ki‐67 and MMP‐2 of tumour sections from different treated groups. Significant differences were indicated as *P ≤ .05; **P ≤ .01; ***P ≤ .001

To determine whether the anti‐tumour and anti‐metastasis effect of BA in vivo is consistent with its effects in vitro, the tumour mouse model of GC cells of the SNU‐16 cell line was established. As displayed in Figure 4A‐C, compared with the control group, BA treatment significantly hindered the tumour growth rate and led to the reduction of tumour weight in vivo. The lungs were isolated and metastatic nodules were quantified. Many large metastatic nodules were observed in the lung tissues of the control group, whereas in the BA‐treatment (40 mg/kg) group, the number of metastatic nodules was significantly reduced (Figure 4D). Furthermore, lung weight of the BA‐treatment group was lower than that of the control group (Figure 4E). In addition, histopathological analysis showed few metastatic tumour cells in the lung sections of BA‐treated mice, implying that BA inhibits pulmonary metastasis of SNU‐16 cells (Figure 4F). Overall, the results suggest that BA suppresses the metastatic activity of GC cells in vivo. As evident from the results of the IHC analysis, the proportions of Ki‐67‐positive and MMP2‐positive cells were significantly lower in the tumour sections of the BA‐treated group than those in the sections of the control group (Figure 5A,B). Taken together, the results show that BA impairs gastric tumour growth in vivo, consistent with our results obtained in vitro.

## 4 DISCUSSION

GC is one of the most prevalent types of malignancies.^1^ GC is a malignancy with high morbidity and mortality, and is the third leading cause of cancer‐related mortality worldwide.^2^, ^3^ Therefore, the need for new and effective anti‐GC drug treatments is urgent. Even though chemical drugs such as Dox and PTX can effectively destroy the DNA in tumour cells, severe side effects are inevitable.^31^ Natural products from plants and animals exhibit high efficiency, low toxicity and other benefits.^32^ It is widely reported that BA is cytotoxic to various types of human cancer cells.^28^, ^29^, ^30^ In the present study, we assessed the anti‐tumour effect of BA on human GC cells in vitro and in vivo. Firstly, we performed MTT and cloning efficiency experiments to determine the cytotoxicity and anti‐proliferation efficacy of BA with regard to GC cells of the SNU‐16 and NCI‐N87 cell lines. The results indicated that BA had significant cytotoxic and proliferation‐inhibitory effects on GC cells. To further study the intrinsic mechanism of apoptosis of SNU‐16 cells due to BA, flow cytometric analysis was carried out. We found that BA induced apoptosis in SNU‐16 cells. However, the proportion of apoptosis was low. The cell viability and colony formation results suggest that BA significantly suppressed these abilities of the SNU‐16 cells. There may be other anti‐tumour mechanisms of BA in cancer therapy. Liu et al demonstrated that BA induces autophagy‐mediated apoptosis through suppression of the PI3K/AKT/mTOR signalling pathway and inhibits hepatocellular carcinoma.^33^ Lewinska A showed that BA‐mediated changes in glycolytic pathway promote cytotoxic autophagy and apoptosis in phenotypically different breast cancer cells.^34^ Furthermore, BA proved to possess immunomodulatory activity by producing pro‐inflammatory cytokines and activation of macrophages.^35^ Therefore, the other anti‐tumour mechanisms of BA should be further investigated in future.

In addition, the migration, invasion and EMT process of cancer cells are the important and key steps in the distant metastatic cascade of cancer cells. BA inhibited the migratory and invasive abilities of SNU‐16 cells. Western‐blot analysis showed that BA suppressed GC cell migration and invasion by impairing the EMT process. Furthermore, our in vivo results showed that BA can distinctly hinder tumour growth, which is consistent with the cytotoxic and apoptotic effects observed in vitro. Other studies demonstrated that BA suppresses breast cancer metastasis by targeting GRP78‐mediated glycolysis and ER stress apoptotic pathway.^36^ Similarly, it is reported that BA inhibits stemness and EMT of pancreatic cancer cells via activation of AMPK signalling.^37^ Furthermore, Nuclear Factor‐κB is responsible for anti‐proliferation and anti‐migration of BA in Urothelial Tumorigenesis.^38^ In conclusion, we assessed the anti‐tumour effect of BA on human GC cells by conducting in vitro and in vivo experiments and found that BA induced apoptosis and suppressed the migration and invasion of GC cells. Overall, the present study shows that BA might serve as an anti‐tumour drug for GC therapy.

## ETHICS STATEMENT

All of the animal experiments in this study were performed according to the National Institutes of Health (Bethesda, MD, USA) guidelines and were approved by the Ethical Committee of First Affiliated Hospital of Gannan Medical College (Ganzhou, China).

## CONFLICT OF INTEREST

The authors declare no conflicts of interest.

## AUTHOR CONTRIBUTIONS

Yun Chen and Yun Zhou designed the experiments; Xiongjian Wu and Chi Liu were involved in performing the designed experiments; Chi Liu and Yun Zhou were responsible for analysing the data and writing and approving the manuscript.

## Data Availability

The data sets used or analyzed in this study are available from the corresponding author on reasonable request.

## References

[1] Global Burden of Disease Cancer C , Fitzmaurice C , Allen C , et al. Global, regional, and National Cancer Incidence, mortality, years of life lost, years lived with disability, and disability‐adjusted life‐years for 32 cancer groups, 1990 to 2015: a systematic analysis for the global burden of disease study. JAMA Oncol. 2017;3(4):524‐548. 27918777 10.1001/jamaoncol.2016.5688 PMC6103527

[2] Tan P , Yeoh KG . Genetics and molecular pathogenesis of gastric adenocarcinoma. Gastroenterology. 2015;149(5):1153‐1162.e3. 26073375 10.1053/j.gastro.2015.05.059

[3] Chen W . Cancer statistics: updated cancer burden in China. Chin J Cancer Res. 2015;27(1):1. 25717219 10.3978/j.issn.1000-9604.2015.02.07 PMC4329178

[4] Xu Y , Zhang G , Zou C , et al. Long non‐coding RNA LINC01225 promotes proliferation, invasion and migration of gastric cancer via Wnt/beta‐catenin signalling pathway. J Cell Mol Med. 2019;23:7581‐7591. 31460694 10.1111/jcmm.14627 PMC6815774

[5] Ghasabi M , Majidi J , Mansoori B , et al. The effect of combined miR‐200c replacement and cisplatin on apoptosis induction and inhibition of gastric cancer cell line migration. J Cell Physiol. 2019;234(12):22581–22592. 31111481 10.1002/jcp.28823

[6] Huang B , Chang C , Wang BL , Li H . ELK1‐induced upregulation of lncRNA TRPM2‐AS promotes tumor progression in gastric cancer by regulating miR‐195/HMGA1 axis. J Cell Biochem. 2019;120(10):16921–16933. 31104318 10.1002/jcb.28951

[7] Yu J , Huang C , Sun Y , et al. Effect of laparoscopic vs open distal gastrectomy on 3‐year disease‐free survival in patients with locally advanced gastric cancer: the CLASS‐01 randomized clinical Trial. JAMA. 2019;321(20):1983‐1992. 31135850 10.1001/jama.2019.5359 PMC6547120

[8] Liu K , Qin M , Huang J . The prescreening tool for gastric cancer in China. Gut. 2019; https://doi: 10.1136/gutjnl‐2019‐319591. 10.1136/gutjnl-2019-319591 31481546

[9] Kim HS , Lee H , Jeung HC , et al. Advanced detection of recent changing trends in gastric cancer survival: up‐to‐date comparison by period analysis. Jpn J Clin Oncol. 2011;41(12):1344‐1350. 22128316 10.1093/jjco/hyr153

[10] Cunningham D , Allum WH , Stenning SP , et al. Perioperative chemotherapy versus surgery alone for resectable gastroesophageal cancer. N Engl J Med. 2006;355(1):11‐20. 16822992 10.1056/NEJMoa055531

[11] Macdonald JS , Smalley SR , Benedetti J , et al. Chemoradiotherapy after surgery compared with surgery alone for adenocarcinoma of the stomach or gastroesophageal junction. N Engl J Med. 2001;345(10):725‐730. 11547741 10.1056/NEJMoa010187

[12] Brachtendorf S , El‐Hindi K , Grosch S . Ceramide synthases in cancer therapy and chemoresistance. Prog Lipid Res. 2019;74:160‐185. 30953657 10.1016/j.plipres.2019.04.002

[13] Grassberger C , Ellsworth SG , Wilks MQ , Keane FK , Loeffler JS . Assessing the interactions between radiotherapy and antitumour immunity. Nat Rev Clin Oncol. 2019;16:729‐745. 31243334 10.1038/s41571-019-0238-9 PMC13492067

[14] Alakurtti S , Makela T , Koskimies S , Yli‐Kauhaluoma J . Pharmacological properties of the ubiquitous natural product betulin. Eur J Pharm Sci. 2006;29(1):1‐13. 16716572 10.1016/j.ejps.2006.04.006

[15] Fulda S , Kroemer G . Targeting mitochondrial apoptosis by betulinic acid in human cancers. Drug Discov Today. 2009;14(17–18):885‐890. 19520182 10.1016/j.drudis.2009.05.015

[16] Tyagi M , Begnini F , Poongavanam V , Doak BC , Kihlberg J . Drug syntheses beyond the rule of 5. Chemistry. 2019;26:49‐88. 31483909 10.1002/chem.201902716

[17] Li Y , Li PK , Roberts MJ , Arend RC , Samant RS , Buchsbaum DJ . Multi‐targeted therapy of cancer by niclosamide: a new application for an old drug. Cancer Lett. 2014;349(1):8‐14. 24732808 10.1016/j.canlet.2014.04.003 PMC4166407

[18] Sun W , Luan S , Qi C , et al. Aspulvinone O, a natural inhibitor of GOT1 suppresses pancreatic ductal adenocarcinoma cells growth by interfering glutamine metabolism. Cell Commun Signal. 2019;17(1):111. 31470862 10.1186/s12964-019-0425-4 PMC6717386

[19] Aiello P , Consalvi S , Poce G , et al. Dietary flavonoids: Nano delivery and nanoparticles for cancer therapy. Semin Cancer Biol. 2019; https://doi: 10.1016/j.semcancer.2019.08.029. 10.1016/j.semcancer.2019.08.029 31454670

[20] Edeline J , Blanc JF , Campillo‐Gimenez B , et al. Prognostic scores for sorafenib‐treated hepatocellular carcinoma patients: a new application for the hepatoma arterial embolisation prognostic score. Eur J Cancer. 2017;86:135‐142. 28987770 10.1016/j.ejca.2017.08.036

[21] Heinzen M , Mettler S , Coradi A , Boutellier R . A new application of value‐stream mapping in new drug development: a case study within Novartis. Drug Discov Today. 2015;20(3):301‐305. 25448754 10.1016/j.drudis.2014.10.009

[22] Liu CM , Qi XL , Yang YF , Zhang XD . Betulinic acid inhibits cell proliferation and fibronectin accumulation in rat glomerular mesangial cells cultured under high glucose condition. Biomed Pharmacother. 2016;80:338‐342. 27133074 10.1016/j.biopha.2016.02.040

[23] Kren BT , Unger GM , Abedin MJ , et al. Preclinical evaluation of cyclin dependent kinase 11 and casein kinase 2 survival kinases as RNA interference targets for triple negative breast cancer therapy. Breast Cancer Res. 2015;17:19. 25837326 10.1186/s13058-015-0524-0 PMC4344788

[24] Zeng A , Hua H , Liu L , Zhao J . Betulinic acid induces apoptosis and inhibits metastasis of human colorectal cancer cells in vitro and in vivo. Bioorg Med Chem. 2019;27(12):2546‐2552. 30910472 10.1016/j.bmc.2019.03.033

[25] Mesaik AM , Poh HW , Bin OY , Elawad I , Alsayed B . In vivo anti‐inflammatory, anti‐bacterial and anti‐Diarrhoeal activity of *Ziziphus jujuba* fruit extract. Open Access Maced J Med Sci. 2018;6(5):757‐766. 29875842 10.3889/oamjms.2018.168 PMC5985874

[26] Grymel M , Zawojak M , Adamek J . Triphenylphosphonium analogues of Betulin and Betulinic acid with biological activity: a comprehensive review. J Nat Prod. 2019;82(6):1719‐1730. 31141361 10.1021/acs.jnatprod.8b00830

[27] Yao N , Wang C , Hu N , et al. Inhibition of PINK1/Parkin‐dependent mitophagy sensitizes multidrug‐resistant cancer cells to B5G1, a new betulinic acid analog. Cell Death Dis. 2019;10(3):232. 30850585 10.1038/s41419-019-1470-z PMC6408511

[28] Lee D , Lee SR , Kang KS , et al. Betulinic acid suppresses ovarian cancer cell proliferation through induction of apoptosis. Biomolecules. 2019;9(7):257. 10.3390/biom9070257 PMC6681197 31277238

[29] Cai Y , Zheng Y , Gu J , et al. Betulinic acid chemosensitizes breast cancer by triggering ER stress‐mediated apoptosis by directly targeting GRP78. Cell Death Dis. 2018;9(6):636. 29802332 10.1038/s41419-018-0669-8 PMC5970196

[30] Wang S , Wang K , Zhang C , et al. Overaccumulation of p53‐mediated autophagy protects against betulinic acid‐induced apoptotic cell death in colorectal cancer cells. Cell Death Dis. 2017;8(10):e3087. 28981110 10.1038/cddis.2017.485 PMC5682653

[31] Rossi A , Di Maio M , Chiodini P , et al. Carboplatin‐ or cisplatin‐based chemotherapy in first‐line treatment of small‐cell lung cancer: the COCIS meta‐analysis of individual patient data. J Clin Oncol. 2012;30(14):1692‐1698. 22473169 10.1200/JCO.2011.40.4905

[32] Li T , Wang N , Zhang T , et al. A systematic review of recently reported marine derived natural product kinase inhibitors. Mar Drugs. 2019;17(9):493. 10.3390/md17090493 PMC6780990 31450856

[33] Liu W , Li S , Qu Z , et al. Betulinic acid induces autophagy‐mediated apoptosis through suppression of the PI3K/AKT/mTOR signaling pathway and inhibits hepatocellular carcinoma. Am J Transl Res. 2019;11(11):6952‐6964. 31814899 PMC6895530

[34] Lewinska A , Adamczyk‐Grochala J , Kwasniewicz E , Deregowska A , Wnuk M . Ursolic acid‐mediated changes in glycolytic pathway promote cytotoxic autophagy and apoptosis in phenotypically different breast cancer cells. Apoptosis. 2017;22(6):800‐815. 28213701 10.1007/s10495-017-1353-7 PMC5401707

[35] Yun Y , Han S , Park E , et al. Immunomodulatory activity of betulinic acid by producing pro‐inflammatory cytokines and activation of macrophages. Arch Pharm Res. 2003;26(12):1087‐1095. 14723345 10.1007/BF02994763

[36] Zheng Y , Liu P , Wang N , et al. Betulinic acid suppresses breast cancer metastasis by targeting GRP78‐mediated glycolysis and ER stress apoptotic pathway. Oxidative Med Cell Longev. 2019;2019:1‐15. 10.1155/2019/8781690 PMC6721262 31531187

[37] Sun L , Cao J , Chen K , et al. Betulinic acid inhibits stemness and EMT of pancreatic cancer cells via activation of AMPK signaling. Int J Oncol. 2019;54(1):98‐110. 30365057 10.3892/ijo.2018.4604 PMC6254859

[38] Inoue S , Ide H , Mizushima T , et al. Nuclear factor‐κB promotes urothelial tumorigenesis and cancer progression via cooperation with androgen receptor signaling. Mol Cancer Ther. 2018;17(6):1303‐1314. 29592878 10.1158/1535-7163.MCT-17-0786

